# Transient ST–T Changes During Submaximal Exercise Revealing Anomalous Right Coronary Artery Origin in an Asymptomatic Athlete

**DOI:** 10.1155/cric/1835973

**Published:** 2026-10-23

**Authors:** Vojtěch Illinger, Jan Kovanda, Kryštof Slabý, Michal Procházka, Jiří Radvanský, Ondřej Materna, Jan Marek

**Affiliations:** ^1^ Department of Rehabilitation and Sports Medicine Second Faculty of Medicine Charles University in Prague and Motol and Homolka University Hospital Prague Czech Republic; ^2^ Children′s Heart Centre Second Faculty of Medicine Charles University in Prague and Motol and Homolka University Hospital Prague Czech Republic; ^3^ Great Ormond Street Hospital for Children Institute of Cardiovascular Sciences at UCL London UK nhs.uk

**Keywords:** anomalous aortic coronary artery origin, athlete, case report, echocardiography, preparticipation screening, stress testing

## Abstract

**Background:**

Anomalous aortic origin of a coronary artery is a recognised structural cause of sudden cardiac death in athletes younger than 35 years. Most affected individuals are asymptomatic prior to cardiac events, and resting and exercise electrocardiography (ECG) findings are frequently normal or non‐specific, limiting the effectiveness of preparticipation screening.

**Case Summary:**

We report a case of a 22‐year‐old asymptomatic athlete in whom preparticipation screening revealed transient ST‐segment depression and T‐wave inversions during submaximal exercise and recovery, whereas findings at peak exercise remained normal. Point‐of‐care echocardiography performed by a sports medicine specialist raised suspicion of an anomalous origin of the right coronary artery from the left aortic sinus of Valsalva, which was subsequently confirmed by computed tomography coronary angiography. High‐risk anatomical features included an acute ostial angle and an interarterial course of the right coronary artery. Functional testing with exercise myocardial stress scintigraphy showed no inducible perfusion defect. Following multidisciplinary consultation, a conservative management strategy with activity modification and close follow‐up was adopted.

**Discussion:**

This case highlights the potential clinical significance of transient ST–T abnormalities occurring during submaximal exercise and recovery, even in the absence of symptoms or abnormalities at peak workload. In this patient, recognition of this reproducible but non‐specific ECG pattern prompted further structural evaluation leading to the diagnosis of AAOCA. It also underscores the limitations of current guidelines in managing asymptomatic athletes with high‐risk coronary anatomy but without evidence of inducible ischaemia, supporting the need for individualised decision‐making.

## 1. Introduction

Anomalous aortic origin of a coronary artery (AAOCA) is one of the most common structural causes of sudden cardiac death (SCD) in young athletes, as reported in several cohorts [1–4]. The prevalence in the general adolescent population is estimated to be approximately 0.44% [5].

AAOCA encompasses several anatomical variants, including origin of the left main coronary artery from the right aortic sinus of Valsalva (associated with a higher risk of cardiac events), origin of the right coronary artery from the left aortic sinus of Valsalva (with limited data regarding prognosis and management) and less common variants such as anomalous origin of the left anterior descending or circumflex artery, inverted coronary arteries and single coronary artery [6].

Clinical presentation is variable. Symptoms may include chest pain or syncope; however, SCD is frequently the first manifestation. In a retrospective study of 27 exercise‐related sudden deaths in young athletes with AAOCA, 55.6% of individuals had no prior cardiovascular symptoms, and only 37.0% reported warning symptoms such as syncope or chest pain before SCD [7].

Resting ECG is usually normal, although it may occasionally show non‐specific changes suggestive of ischaemia [8]. Exercise ECG may reveal ST–T abnormalities, ventricular arrhythmias or only non‐specific repolarisation changes, but it is often completely normal, limiting its diagnostic value.

Early diagnosis may be achieved using transthoracic echocardiography in young athletes; however, its sensitivity is highly operator‐dependent [8–10]. When AAOCA is suspected, definitive diagnosis is established using computed tomography coronary angiography or cardiac magnetic resonance imaging [11, 12].

Clinical risk factors for SCD include age under 35 years, vigorous physical activity and the presence of symptoms. High‐risk anatomical features include an acute ostial angle with a slit‐like orifice, high take‐off and an interarterial or intramural course of the coronary artery, all of which are associated with an increased risk of adverse cardiac events [13]. Advanced imaging techniques, including three‐dimensional reconstruction and virtual modelling, further enhance anatomical assessment [14].

Emerging methods for risk stratification, such as fractional flow reserve and intravascular ultrasound, are currently under investigation [15]. Functional nonpharmacological stress imaging is recommended to assess the presence of inducible myocardial ischaemia [12].

Management depends on patient age, level of physical activity, symptoms, coronary anatomy, presence of high‐risk anatomical features and evidence of inducible ischaemia. Treatment options include observation, restriction of physical activity or surgical intervention. Current surgical strategies include coronary unroofing, pulmonary artery translocation and coronary reimplantation [16].

According to current ESC guidelines, participation in moderate‐ to high‐intensity competitive sports is not recommended in individuals younger than 40 years with high‐risk features, regardless of symptoms (Class III, Level of evidence C). Surgical intervention is recommended in symptomatic patients with evidence of stress‐induced ischaemia or high‐risk anatomy (Class I, Level of evidence C) [13]. Following successful surgical repair, return to competitive sports may be considered after at least 3 months, provided the patient is asymptomatic and no inducible ischaemia or arrhythmia is present on cardiopulmonary exercise testing (Class IIb, Level of evidence C) [17]. However, evidence supporting surgical intervention in asymptomatic individuals remains limited.

A significant gap persists in current guidelines regarding the management of asymptomatic patients with anomalous origin of the right coronary artery from the left aortic sinus of Valsalva who exhibit high‐risk anatomical features but no inducible myocardial ischaemia. In contrast, expert consensus from the American Association for Thoracic Surgery supports continued athletic participation following appropriate counselling (Class IIa, Level of evidence C) [16]. Similarly, recent recommendations from the American Heart Association and American College of Cardiology consider competitive sports participation reasonable within a shared decision‐making framework, with ongoing clinical follow‐up [17].

This case illustrates the potential diagnostic relevance of transient ST–T abnormalities during submaximal exercise, a pattern that remains underrecognised in current screening strategies.

## 2. Case Presentation

A 22‐year‐old male athlete was referred to a sports medicine department for preparticipation screening prior to commencing university studies in physical education. His family history was negative for cardiovascular disease. His medical history was unremarkable, apart from juvenile glaucoma treated with topical brinzolamide. He attended regular general practitioner follow‐up and had no risk factors for premature atherosclerosis. He did not consume alcohol, denied the use of performance‐enhancing substances, was a lifelong non‐smoker and remained physically active, engaging primarily in calisthenics and gymnastics for approximately 2 h per day, five times per week.

At an initial evaluation 1 year earlier, transient non‐specific T‐wave inversions had been observed during exercise testing. A subsequent transthoracic echocardiogram was reported as normal, and the patient was cleared for academic participation.

At current presentation, he remained asymptomatic, denying syncope, chest pain, palpitations or dyspnoea. Physical examination was unremarkable. Anthropometric measurements (79.5 kg, 182 cm, body mass index 23.8 kg/m^2^) were consistent with an athletic build. Heart rhythm was regular without murmurs, lung auscultation was normal, blood pressure was 126/70 mmHg and resting oxygen saturation was 97%. Resting 12‐lead electrocardiography demonstrated sinus rhythm with early repolarisation in the lateral leads and voltage criteria consistent with physiological left ventricular hypertrophy (Figure 1).

**Figure 1:**
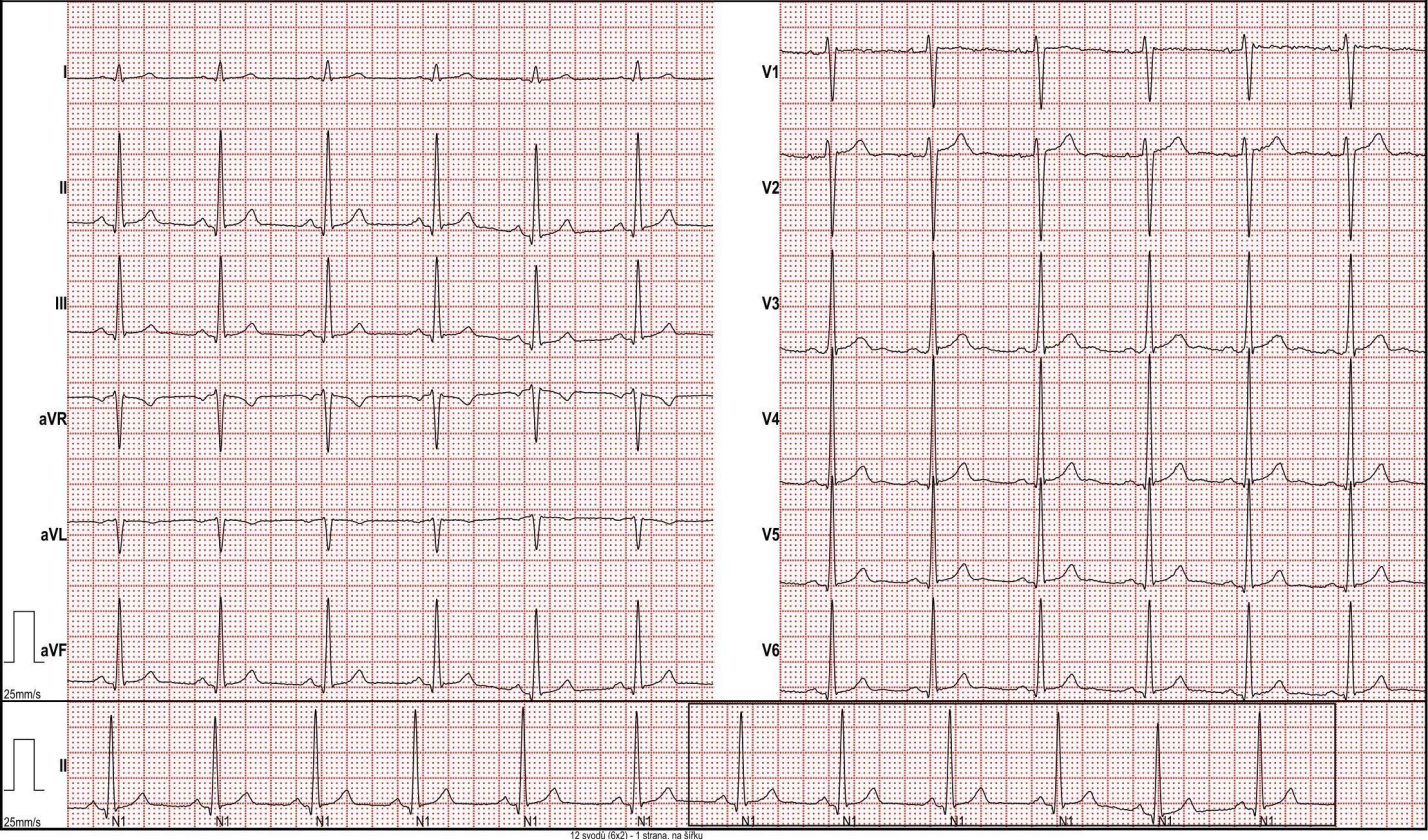
Resting 12‐lead electrocardiogram (25 mm/s, 1 mV/cm).

Despite a low pretest probability of coronary artery disease, an incremental exercise test on a bicycle ergometer was performed in accordance with regional screening protocols. The patient achieved 300 W (3.8 W/kg, 97.4% predicted) after 11 min of exercise, with termination because of maximal perceived exertion (Borg scale 20/20). The haemodynamic response was normal, with a peak systolic blood pressure of 200 mmHg and a nadir oxygen saturation of 96%. No symptoms were reported during testing.

The exercise ECG demonstrated a gradual increase in sinus rhythm, reaching a peak heart rate of 192 bpm (98.9% of predicted maximum), with appropriate recovery thereafter. No significant arrhythmias were observed, apart from a single ventricular couplet immediately after exercise cessation. Notably, a reproducible pattern of transient 0.10 mV ST‐segment depression with T‐wave inversion was observed in the inferolateral leads during submaximal exercise (heart rate 130–150 bpm). These changes resolved at higher workloads (≥ 160 bpm) and remained absent at peak exercise. This atypical pattern, with abnormalities present at submaximal but not peak workload, was considered abnormal and prompted further cardiovascular evaluation. During recovery, similar ST–T abnormalities reappeared and gradually normalised as the heart rate decreased to 100 bpm (Figure 2). Although non‐specific, the reproducibility of these changes warranted further investigation in this young athlete.

**Figure 2:**
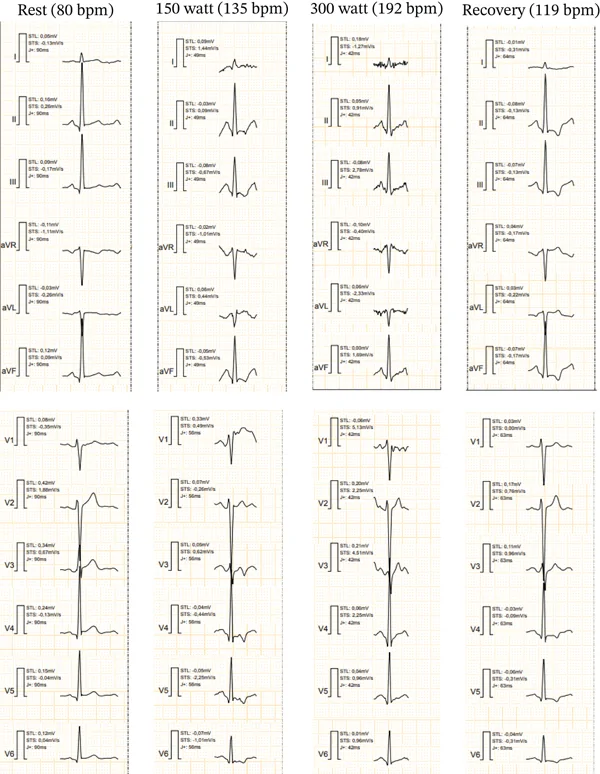
Exercise electrocardiogram (10 consecutive signal‐averaged QRS complexes) at rest, during incremental exercise (submaximal and peak heart rate) and during recovery. bpm, beats per minute.

Laboratory testing excluded secondary causes. Complete blood count was normal, high‐sensitivity troponin I was not elevated (< 2 ng/L; upper limit of normal 34.2 ng/L), NT‐proBNP was within normal limits (< 35 ng/L), and lipid profile, including lipoprotein(a), was unremarkable.

Point‐of‐care echocardiography was performed using the SHARP (Screening the Heart of the Athlete Research Program) protocol by a sports medicine physician with focused echocardiography training. The origin of the right coronary artery was not visualised in the right coronary sinus, raising suspicion of an anomalous course (Figure 3). This finding was subsequently confirmed by an experienced cardiac sonographer, and the patient was referred for computed tomography coronary angiography.

**Figure 3:**
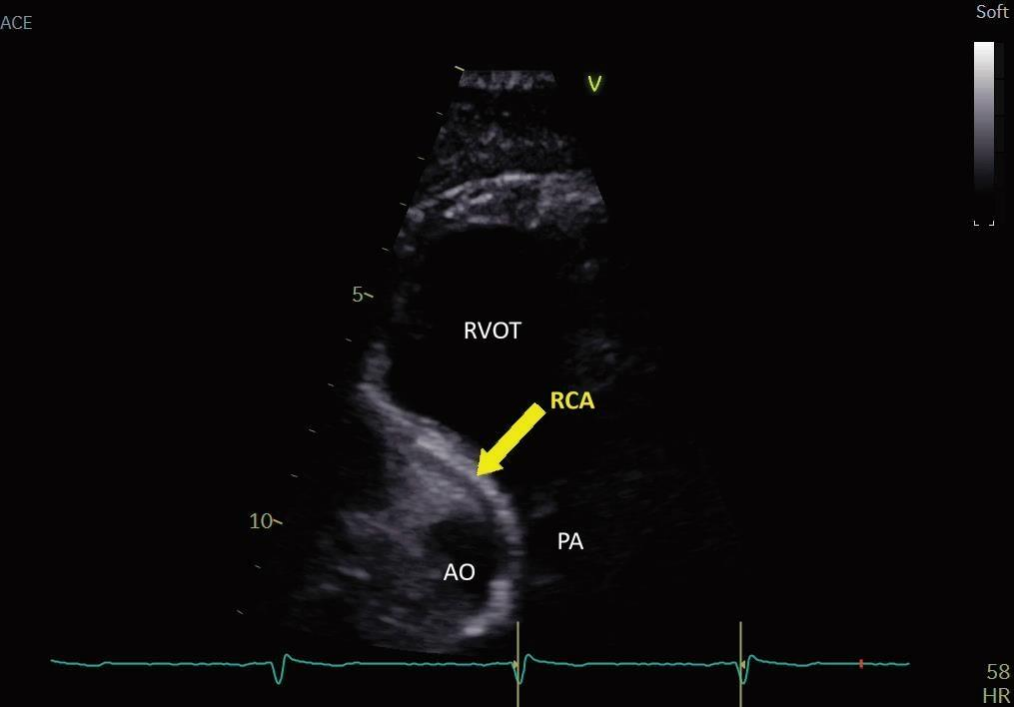
Transthoracic echocardiography, parasternal short‐axis view. Yellow arrow indicates the suspected course of the right coronary artery (RCA). AO, aorta; RVOT, right ventricular outflow tract; PA, pulmonary artery.

Computed tomography coronary angiography with three‐dimensional reconstruction demonstrated an anomalous origin of the right coronary artery from the left coronary sinus, with an acute ostial angle and an interarterial course (Figures 4 and 5).

**Figure 4:**
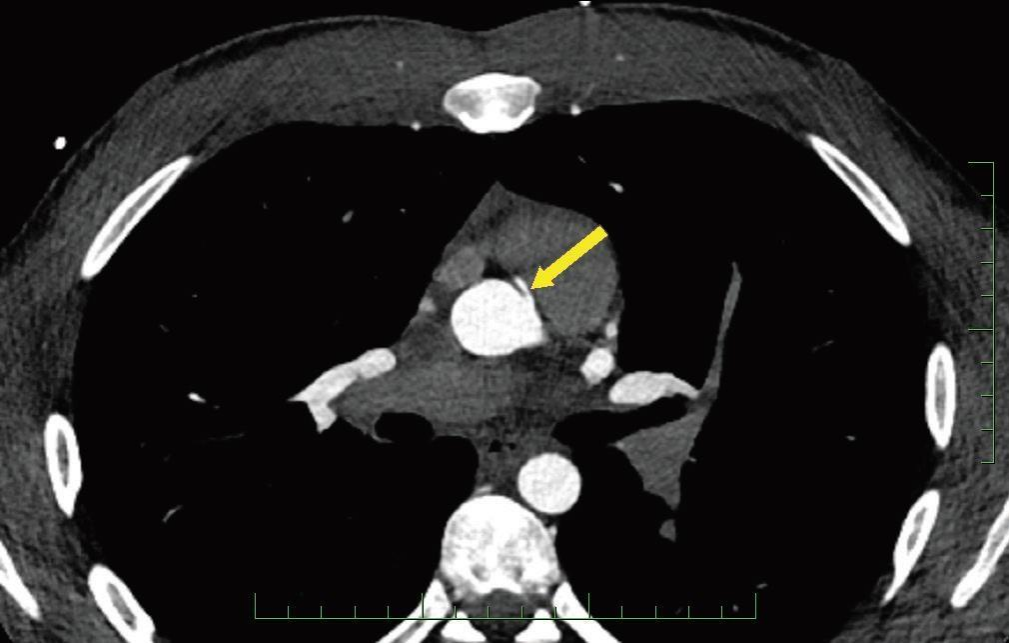
Computed tomography coronary angiography using a prospective ECG‐triggered, high‐pitch turbo FLASH acquisition during the diastolic phase with contrast enhancement. Yellow arrow indicates anomalous origin of the right coronary artery from the left coronary cusp, with an acute ostial angle and interarterial course.

**Figure 5:**
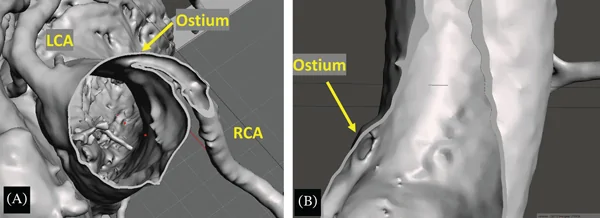
Three‐dimensional reconstruction images demonstrating high‐risk anatomical features: (A) acute ostial angle with proximal narrowing and (B) slit‐like orifice of the right coronary artery. RCA, right coronary artery; LCA, left coronary artery. Yellow arrows indicate the RCA ostium.

Ambulatory 24‐h ECG monitoring performed during the patient′s usual training routine did not reveal significant arrhythmias or dynamic ST–T abnormalities. Functional assessment with exercise myocardial scintigraphy (275 W, 185 bpm) reproduced the ECG changes but showed no evidence of an inducible perfusion defect (Figure 6).

**Figure 6:**
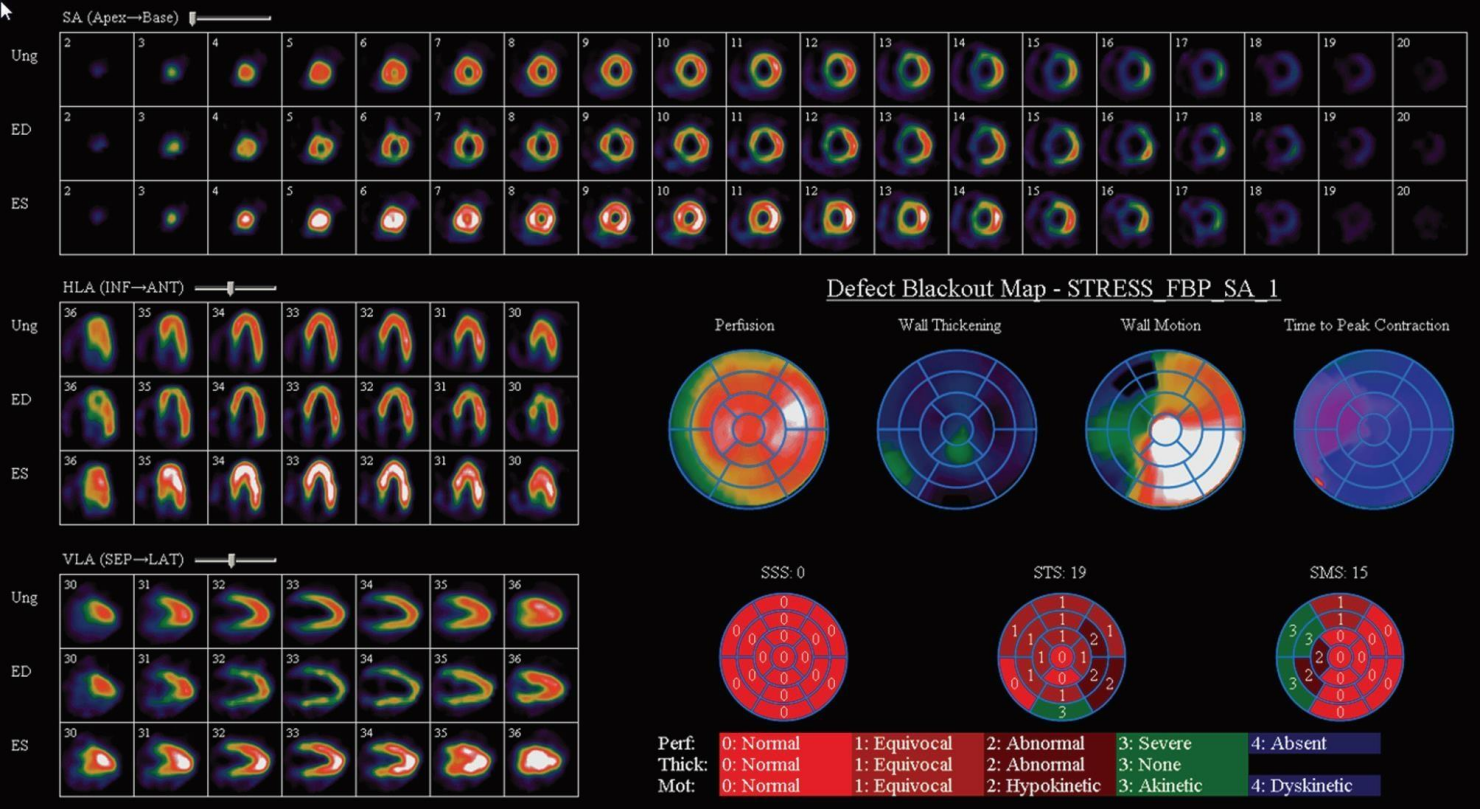
Myocardial stress scintigraphy demonstrating no evidence of an inducible perfusion defect.

Following completion of the diagnostic workup, the patient was referred to a tertiary cardiac centre, where surgical revascularisation (RIMA–RCA bypass) was recommended because of the anatomical features. Given the absence of symptoms, the patient sought a second opinion at another tertiary centre, where a conservative approach with cessation of competitive sports was advised.

Because of conflicting recommendations, a superconsultation with a European expert in coronary anomalies was obtained. A conservative management strategy was confirmed, and following shared decision‐making, the patient was allowed to continue professional activity as a personal trainer with activity modification. He was counselled regarding warning symptoms, provided with an emergency action plan, and advised on access to an automated external defibrillator [18]. Annual follow‐up with functional testing was recommended. Surgical intervention (e.g., ostial or pulmonary artery translocation) was reserved for the development of symptoms or evidence of inducible ischaemia [13, 16].

The patient subsequently completed his studies and currently works as a personal fitness coach without reported complications.

## 3. Discussion

Current consensus regarding preparticipation cardiovascular screening in athletes includes personal and family history, physical examination and resting electrocardiography [19]. There is no universal agreement on the routine use of exercise testing for screening purposes. However, in several European countries, including Italy and the Czech Republic, exercise testing is commonly incorporated into screening protocols to assess fitness and detect exercise‐induced ischaemia or arrhythmias [20]. This practice frequently results in non‐specific ECG abnormalities, which may be challenging to interpret and often necessitate further diagnostic evaluation or longitudinal follow‐up.

Malhotra et al. [21] demonstrated that the addition of echocardiography to large‐scale preparticipation screening improves the detection of structural abnormalities, including bicuspid aortic valve disease and anomalous coronary artery origin. In the present case, point‐of‐care echocardiography performed by a trained sports medicine physician raised early suspicion of a coronary anomaly, subsequently confirmed by advanced imaging. This supports the feasibility and potential value of incorporating focused echocardiographic assessment into athlete screening programmes. Importantly, point‐of‐care echocardiography is operator‐dependent and should not be considered definitive for the diagnosis or exclusion of coronary anomalies. Its value in this case was to trigger expert echocardiographic reassessment and subsequent computed tomography coronary angiography, which established the anatomy. Structured protocols and appropriate training may nevertheless allow point‐of‐care echocardiography to serve as a useful adjunct to athlete evaluation in selected settings [18, 22]. The use of structured imaging protocols tailored to young athletes is essential to minimise the risk of missing structural conditions associated with SCD, including congenital heart disease, cardiomyopathies, aortic pathology and coronary anomalies [22]. This case also illustrates that coronary origin may be overlooked if it is not systematically assessed during echocardiographic evaluation.

A key feature of this case was the reproducible pattern of ST–T abnormalities during submaximal exercise and recovery, with normal findings at peak workload. This atypical presentation differs from classical exercise‐induced ischaemia, and its underlying mechanism remains uncertain. Dynamic coronary compression or altered haemodynamics associated with anomalous coronary anatomy represent possible explanations; however, the absence of inducible perfusion abnormalities does not support interpreting these changes as evidence of myocardial ischaemia. Importantly, such transient and non‐specific ECG changes may be difficult to interpret in asymptomatic athletes and may warrant further evaluation when reproducible or otherwise clinically unexplained.

Management in this patient was particularly challenging because anatomical and functional risk markers were discordant. The interarterial course, acute ostial angle, proximal narrowing and slit‐like orifice raised concern, whereas the patient remained asymptomatic and exercise myocardial scintigraphy showed no inducible perfusion defect. Current recommendations do not provide unequivocal guidance for all asymptomatic patients with anomalous right coronary artery origin and high‐risk anatomical features, and both surgical and conservative strategies may therefore be considered according to the individual clinical context [13, 16, 17]. Surgical correction remains an important option when symptoms, demonstrable ischaemia or other compelling high‐risk features are present, and several surgical techniques, including coronary reimplantation, may be selected according to the specific anatomy [23]. In the present case, after multidisciplinary assessment and external expert consultation, the absence of symptoms and demonstrable inducible ischaemia was considered to weigh against immediate intervention. Conservative management was therefore selected through individualised shared decision‐making, incorporating activity modification, education regarding warning symptoms, emergency planning and close longitudinal follow‐up. This approach should not be extrapolated to all asymptomatic athletes with AAOCA, for whom management requires case‐by‐case assessment of anatomy, functional findings, sporting exposure and patient preferences.

## 4. Clinical Implications

This case highlights that transient ST–T abnormalities occurring during submaximal exercise or recovery should not be routinely dismissed as benign in young athletes and may warrant further cardiovascular evaluation. In this patient, recognition of this pattern contributed to the diagnostic pathway leading to identification of AAOCA.

## Funding

This study was funded by the Ministerstvo Zdravotnictví Ceské Republiky (10.13039/501100003243).

## Consent

The authors confirm that a written consent for the submission and publication of this case report was obtained from the patient in accordance with COPE guidelines.

## Conflicts of Interest

The authors declare no conflicts of interest.

## Data Availability

The data that support the findings of this study are available on request from the corresponding author. The data are not publicly available due to privacy or ethical restrictions.
